# Prescription of vitamin D was associated with a lower incidence of hip fractures

**DOI:** 10.1038/s41598-023-40259-6

**Published:** 2023-08-09

**Authors:** Mitsutaka Yakabe, Tatsuya Hosoi, Shoya Matsumoto, Kenji Fujimori, Junko Tamaki, Shinichi Nakatoh, Shigeyuki Ishii, Nobukazu Okimoto, Kuniyasu Kamiya, Masahiro Akishita, Masayuki Iki, Sumito Ogawa

**Affiliations:** 1 Department of Geriatric Medicine, Graduate School of Medicine, The University of Tokyo, 7-3-1 Hongo, Bunkyo-ku, Tokyo, 113-8655 Japan; 2 Department of Health Administration and Policy, Tohoku University School of Medicine, 2-1 Seiryo-Machi, Aoba-ku, Sendai, Miyagi 980-8575 Japan; 3 Department of Hygiene and Public Health, Faculty of Medicine, Osaka Medical and Pharmaceutical University, 2-7 Daigakumachi, Takatsuki, Osaka 569-8686 Japan; 4 Department of Orthopedic Surgery, Asahi General Hospital, 477 Tomari, Asahimachi, Shimo-Nikawa-gun, Toyama, 939-0798 Japan; 5 Department of Regulatory Science, School of Pharmacy, Tokyo University of Pharmacy and Life Sciences, 1432-1 Horinouchi, Hachiouji, Tokyo 193-0392 Japan; 6 Okimoto Clinic, 185-4 Kubi, Yutaka-machi, Kure, Hiroshima 734-0304 Japan; 7 Kindai University Faculty of Medicine, 377-2 Oono-Higashi, Osaka-Sayama, Osaka 589-8511 Japan; 8 National Database Japan-Osteoporosis Management (NDBJ-OS) Study Group, Kindai University Faculty of Medicine, 377-2 Oono-Higashi, Osaka-Sayama, Osaka 589-8511 Japan

**Keywords:** Epidemiology, Therapeutics

## Abstract

Patients with osteoporosis are prone to fragility fractures. Evidence of the effects of active forms of vitamin D on hip fracture prevention is insufficient. We examined the association between vitamin D prescription and incidence of new fractures using the data of osteoporotic patients from the nationwide health insurance claims database of Japan. The follow-up period was 3 years after entry. The untreated patients were never prescribed vitamin D during follow-up (n = 422,454), and the treated patients had a vitamin D medication possession ratio of ≥ 0.5 at all time points (n = 169,774). Propensity score matching was implemented on these groups, yielding 105,041 pairs, and subsequently, the control and treatment groups were established and analyzed. The incidence of new fractures was significantly lower in the treatment group compared with the control group (6.25% vs. 5.69%, hazard ratio 0.936 [95% confidence interval 0.904–0.970], *p* < 0.001*). By site, hip fractures significantly decreased (0.89% vs. 0.42%, *p* < 0.001), but not vertebral and radial fractures. Subgroup analysis by vitamin D type showed a significantly lower incidence of total fractures only in alfacalcidol (hazard ratio 0.676 [95% confidence interval 0.628–0.728], *p* < 0.001*). The results suggest that vitamin D prescription was associated with a reduced incidence of hip fractures.

## Introduction

Osteoporosis is a systemic skeletal disease involving bone tissues, leading to bone fragility and susceptibility to fracture^1^. As the world population ages, the number of patients with osteoporosis and resulting fractures is expected to increase^2^.

Studies have reported that 98% of hip fractures^3^ and 100% of wrist fractures were caused by falls^4^. Age-related sarcopenia, frailty, physical deterioration, and various concomitant diseases increase the risk of falls, which may lead to fractures. Fractures are associated with disability, lower quality of life, and increased risk of refractures and mortality in older people^5–7^. Older women who have experienced initial fractures are at high risk of early and subsequent fractures^8^. Mortality after a hip fracture is very high compared with the general population, with 32.7% of patients dying within 2 years following fracture onset, with pneumonia and circulatory diseases as the most common causes of death^9^. Therefore, osteoporosis treatment and fall prevention are important to prevent fractures in older people.

Vitamin D is used to treat osteoporosis, and activated vitamin D has been reported to increase bone mineral density (BMD)^10, 11^. A meta-analysis showed that low serum 25(OH)D level is associated with a higher risk of hip fracture in older people^12^. A systematic umbrella review of meta-analyses of controlled trials of the effects of vitamin D supplementation (with or without calcium [Ca]) have shown that vitamin D reduced the risk of hip and any fractures^13^. In another meta-analysis, active vitamin D preparations significantly reduced the incidence of vertebral and nonvertebral fractures compared with natural vitamin D supplementation^14^. Another systematic review reported that alfacalcidol (ALF) and calcitriol (CAL) significantly reduced the incidence of nonvertebral fractures, and subgroup analyses demonstrated a significant reduction of the incidence of vertebral fractures with ALF, but not with CAL^15^. Eldecalcitol (ELD), a derivative of CAL, is a more potent inhibitor of bone resorption, having potent bone mass-increasing effects^16^. Some studies have shown that ELD is more effective than ALF in increasing BMD and preventing bone fractures^17–19^. In a meta-analysis, ELD significantly increased lumbar BMD, total hip BMD, and femoral neck BMD and significantly reduced the incidence of vertebral fractures compared with ALF, but did not reduce the incidence of nonvertebral fractures^20^.

Vitamin D might also play an important role in skeletal muscles. In animal experiments, myocyte-specific VDR knockout mice showed decreased expressions of genes related to cell cycle progression and Ca regulation in skeletal muscles and reduced lean mass, grip strength, and running speed^21^. In humans, vitamin D has been reported to prevent falls. In a meta-analysis, supplemental vitamin D and active forms of vitamin D reduced falls in older people^22^. Vitamin D3 reduced the rate of falls in a randomized-controlled trial (RCT) of postmenopausal women aged ≤ 65 years with osteoporosis^23^. These effects of preventing falls might be involved in the reduction of fracture risk.

Although vitamin D supplementation and active forms of vitamin D have been suggested to increase BMD and reduce the risk of vertebral and nonvertebral fractures, few studies have focused on the prevention of hip fractures.

In this study, we examined the association between the presence or absence of vitamin D prescriptions and the incidence of osteoporosis-related bone fractures, using data from the National Database in Japan.

## Methods

Herein, “vitamin D” refers to ALF, CAL, and ELD. “Osteoporosis-related fractures” refers to hip, vertebral, and radial fractures.

### Data source

The Japanese health insurance system are described elsewhere^24^. Japan has a universal coverage of social health insurance and Japanese inhabitants are obliged to join one of the three sub-systems; National Health Insurance (for self-employment), Society Health Insurance (for employee) and Special scheme for the aged (75 years old or over). At the end of each month, medical facilities send sets of claims for reimbursement to insurers via the review organization. For that purpose, medical facilities use a special computer system, in which all procedures, drugs and devices for reimbursement are registered for each patient by daily basis. There is a standard code for each of all procedures, drugs and devices.

The Japanese Ministry of Health, Labor and Welfare (MHLW) have developed the National Database of Health Insurance Claims and Specific Health Checkups of Japan since 2008^24^. The National Database (NDB) registers all healthcare insurance claims and detailed datasets at the individual patient base: that is, the database covers all the data of the Japanese inhabitants. The NDB contains the detailed information such as insurer’s code, insured ID number, diagnoses, age, sex, date of consultation for out-patient service, procedures, and drugs provided with information of date, etc. More than 1,700 million records are registered into the NDB every year. The NDB can be used for research with the approval by the MHLW.

### Study design

This study is a retrospective cohort study using data from the NDB. ID numbers are generated by an encrypting function to make data anonymous but combinable for the same patient. The protocol of this study was approved by the MHLW.

### Patients and data extraction

This study used NDB data from March 2012 to March 2019. Patients for whom 3.5 years of data were available were included in the analysis if they met the inclusion and exclusion criteria (i.e., no one died during the period of 3.5 years).

Data of patients who meet all of the following inclusion criteria were used for analysis in this study: (1) aged > 40 years at the date of entry, (2) diagnosed with osteoporosis as of the date of entry, (3) have never been prescribed vitamin D in the 6 months before the date of entry, and (4) it is known whether they are newly diagnosed as osteoporosis-related fractures or not within 3 years from the date of entry.

Patients were excluded if they met any of the following exclusion criteria: (1) have been prescribed anything other than osteoporosis medications at least once between the date of entry and the end of follow-up, (2) are prescribed medications for more than 90 days at one time (because the maximum number of drugs that can be prescribed per day is 90 in Japan), and (3) developed an osteoporosis-related fracture on the date of entry or within 90 days of the date of entry.

According to these criteria, the following data of the patients were extracted from the NDB: anonymized ID; date of entry; date 1095 days after the date of entry; age at the date of entry; sex; number of ALF, CAL, or ELD prescription days from entry to day 90, from day 91 to day 180, from day 181 to day 365, from day 366 to day 730, and from day 731 to day 1095 after entry; presence or absence of hip, vertebral, or radial fractures from entry to day 1095; and date of diagnosis of such fractures. Patients who met the inclusion and exclusion criteria and had no missing data for 3 years after entry were analyzed. The date of entry ranged from September 2012 to March 2016. There was no entry from March 2012 to August 2012 because data from this period were retrieved only to determine whether patients met the inclusion criteria 3.

The end of follow-up was defined as one of the following: (1) the first date within 3 years of the date of entry, when a new osteoporosis-related fracture was diagnosed, or (2) the date exactly 1095 days (= 3 years) have elapsed since the date of entry (if the patient did not develop a new osteoporosis-related fracture). The last follow-up was completed in March 2019.

The drug and diagnosis codes used to extract data from the NDB are provided as Supplementary File 1. Each drug has a 9-digit code, and each disease has a 7-digit Japanese Standard Disease Code (JSDC). If a patient data had one of the JSDCs of osteoporosis-related fractures and the registration date of the JDCS during the follow-up, the patient was regarded to have developed a new osteoporosis-related fracture on the date.

### Patient selection based on the medication possession ratio (MPR)

The MPR was used to quantify medication adherence. MPR is the sum of days’ supply divided by the number of days in a period^25^. To ensure the effects of vitamin D, only those who maintained an MPR above a certain level throughout the follow-up period were considered in the treatment group. The number of tracking days from the date of entry was divided into five periods: entry to day 90, day 91 to day 180, day 181 to day 365, day 366 to day 730, and day 731 to day 1095. Untreated patients were defined as “those who were never prescribed vitamin D from the date of entry to the end of follow-up”. Treated patients were defined as “those with MPR ≥ 0.5 for all the periods before the end of follow-up”. Patients who did not fit into either group were excluded.

### Propensity score (PS) matching

We performed one-to-one PS matching between the untreated and treated patients^26–28^. To estimate PS, we employed a logistic regression model, where the covariates included age, sex, prescriptions of osteoporosis medications (e.g., BPs, PTH, menatetrenone, and SERMs) during the 6 months before entry, and incident fractures (hips, vertebrae, and radius) during the six months before entry, and place of residence (47 prefectures); the outcome was untreated (coded as 0) or treated (coded as 1). We calculated the C-statistic to assess the model’s discriminatory ability. Using PS estimates, we performed nearest-neighbor matching without replacement, setting the caliper at 0.2 times the standard deviation of the PS estimates^27^. This yielded matched pairs, and the PS-matched control and treatment groups were established.

The incidence rate of osteoporosis-related fractures was compared between the PS-matched control and treatment groups. The rate was also compared by fracture sites.

### Subgroup analysis by the type of vitamin D

The patients in the PS-matched treatment group were stratified based on the type of prescribed vitamin D: the ALF, CAL, ELD groups, and patients prescribed more than one type of vitamin D during follow-up were included in the “mix” group. As a subanalysis, fracture incidence by type of vitamin D was compared in the treatment group. It was also compared with the control group.

### Statistical analyses

Data analysis was performed using R 3.3.3 software (R Foundation for Statistical Computing, Vienna, Austria), and *p* < 0.001* was considered significant. The incidence rates of fractures were analyzed using the Kaplan–Meier curve and Log-rank test. The Pearson χ^2^ test was used for categorical variables, with two-sided.

To evaluate factors on the incidence of new fractures, a Cox regression analysis was performed. We set age, sex, prescriptions before entry, incident fractures before entry, and place of residence as risk-adjusting covariates, the treatment as explanatory variable, and the development of osteoporosis-related fractures as the dependent variable (fracture no = 0, fracture yes = 1).

### Ethics approval and consent to participate

The study protocol was approved by the Institutional Review Board of the Graduate School of Medicine, The University of Tokyo (Approval Number: 2020291NI), as well as the MHLW. All methods were performed in accordance with the relevant regulations and guidelines. The data in this study were completely anonymous; therefore, the need of informed consent was waived by the Institutional Review Board of the Graduate School of Medicine, The University of Tokyo.

## Results

**Figure 1:**
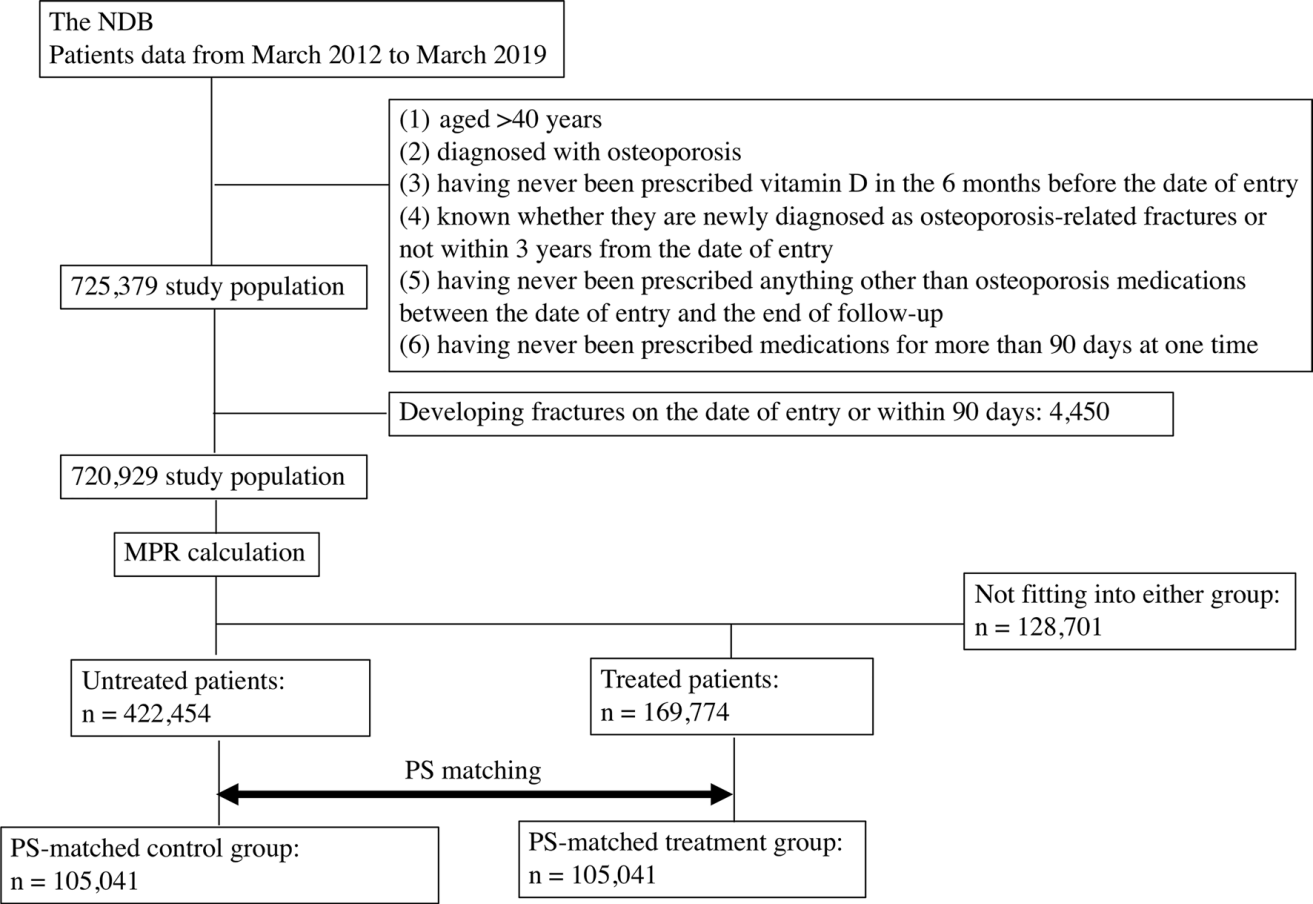
Flow diagram for the patient selection and PS matching. The chart shows how the control group patients were extracted from the NDB. Characteristics of the untreated and treated patients, the PS-matched control and treatment groups are described in Table 1.

The patient selection process is illustrated in Fig. 1. From the patient data from March 2012 to March 2019, a total of 725,379 patients who met the inclusion and exclusion criteria were extracted. Among these patients, 4450 who developed fractures within 90 days of entry were excluded, leaving 720,929 patients. After the patient selection based on the calculated MPR, untreated patients numbered 422,454, and treated patients numbered 169,774. The characteristic of the untreated and treated patients are presented in Table 1, and their place of residence (prefectures) is provided in Supplementary Table 1. Compared to untreated patients, treated patients were significantly older, had a higher proportion of men, had a lower proportion of prescriptions of other osteoporosis drugs (BPs, PTH, Menatetrenone, and SERMs) before entry, and had a higher incidence of fractures (hips and vertebrae) before entry. During the follow-up, 26,845 (6.4%) untreated patients developed fractures (hip, 4008 cases; vertebras, 20,197 cases; radius, 2640 cases), while 8,398 (4.9%) treated patients developed fractures (hip, 687 cases; vertebras, 6730 cases; radius, 981 cases). The incidence of all fractures was significantly lower in treated patients compared to untreated patients (p < 0.001*).

**Table 1:** The characteristic of the patients before and after the PS matching.

	Before PS matching				After PS matching		
	Untreated patients	Treated patients	SMD	*p*-value	PS-matched control group	PS-matched treatment group	SMD
n	422,454	169,774			105,041	105,041	
Age	73.4 ± 9.7	73.7 ± 9.2	0.033	< 0.001*	73.4 ± 9.5	73.4 ± 8.6	0.004
Sex							
Men	18,544 (4.4%)	8465 (5.0%)	0.028	< 0.001*	2456 (2.3%)	2373 (2.3%)	0.005
Women	403,910 (95.6%)	161,309 (95.0%)			102,585 (97.7%)	102,668 (97.7%)	
Prescriptions before entry							
BPs	182,630 (43.2%)	48,927 (28.8%)	0.304	< 0.001*	48,401 (46.1%)	48,927 (46.6%)	0.010
PTH	36,537 (8.6%)	4449 (2.6%)	0.264	< 0.001*	4753 (4.5%)	4449 (4.2%)	0.014
Menatetrenone	11,705 (2.8%)	2372 (1.4%)	0.096	< 0.001*	3418 (3.3%)	2372 (2.3%)	0.061
SERMs	204,561 (48.4%)	53,656 (31.6%)	0.348	< 0.001*	52,831 (50.3%)	53,656 (51.1%)	0.016
Incident fractures before entry							
Hip	5476 (1.3%) in total	3143 (1.9%) in total	0.022	< 0.001*	1913 (1.8%) in total	1709 (1.6%) in total	0.010
Vertebra			0.039	< 0.001*			0.012
Radius			0.003	0.499			< 0.001

Age is expressed as mean ± standard deviation.

To comply with the NDB regulations, only the total number of three types of fractures are presented in the incident fractures before entry.

*SMD* standardized mean difference.

*p < 0.001 is considered significant.

In the logistic regression model, the variance inflation factors were below 5 in all the covariates, indicating no multicollinearity. The C-statistic was 0.7245. After performing PS matching, the PS-matched control group (n = 105,041) and the PS-matched treatment group (n = 105,041) were established. The characteristics of the groups are shown in Table 1, and the patients’ place of residence (prefectures) is provided in Supplementary Table 1. After PS matching, the standardized mean difference (SMD) between the two groups were < 0.1 in all the variables, suggesting that the distributions of patient background variables were well balanced. Incidentally, within the section of incident fractures before entry in Table 1, the specific quantities of hip, vertebra, and radius fractures are not displayed; solely the numbers in total are provided. This practice is implemented to comply with the privacy policy of the NDB that stipulates that the presentation of data is prohibited when the number of fracture events is below ten.

**Figure 2:**
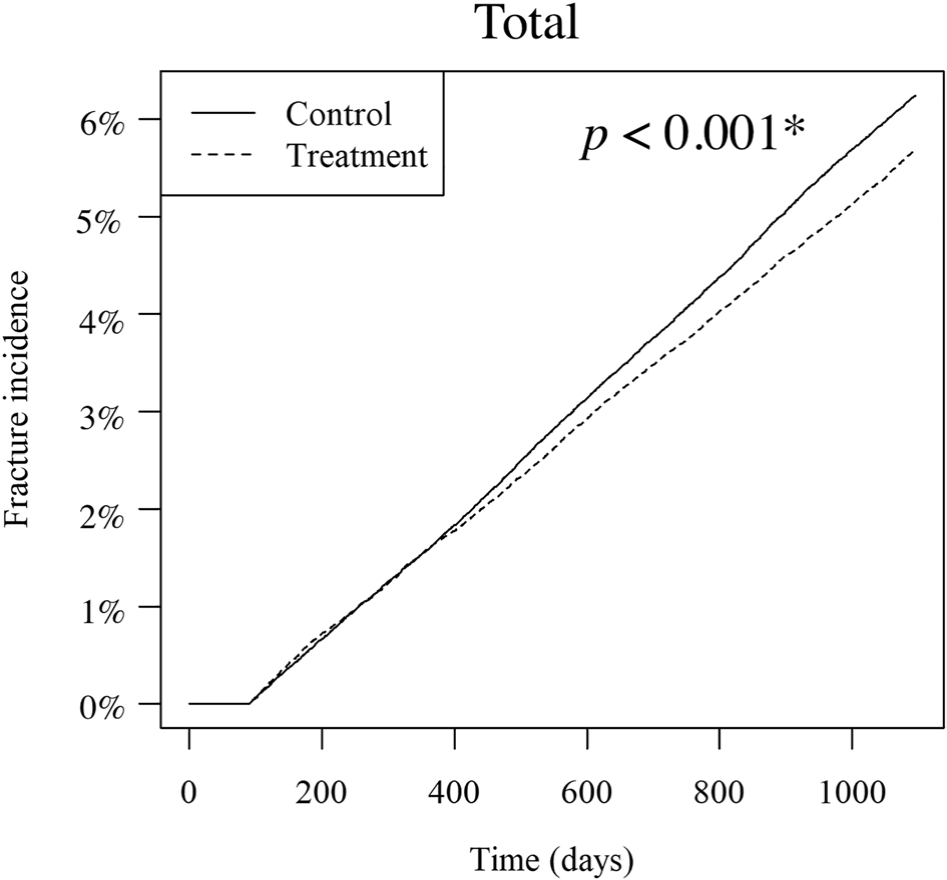
Cumulative incidence of total fractures in the PS-matched control and treatment groups. The solid line represents the PS-matched control group, and the dashed line represents the PS-matched treatment group.

The incidence rates of all osteoporosis-related fractures during the follow-up period were 6.25% (6562 cases) in the PS-matched control group and 5.69% (5974 cases) in the PS-matched treatment group. The Kaplan–Meier curve is presented in Fig. 2. The log-rank test showed that the fracture rate was significantly lower in the PS-matched treatment group (*p* < 0.001*).

To examine whether the treatment independently affected the development of fractures, we conducted a Cox regression analysis in the PS-matched control and treatment groups. The log minus log plots of the control and treatment groups were approximately parallel, suggesting the validity of proportional hazards assumption. After adjustments of age, sex, prescriptions before entry, incident fractures before entry, and place of residence, the treatment significantly reduced the incidence of new fractures (HR 0.936 [95% confidence interval (95% CI) 0.904–0.970], *p* < 0.001*) (Table 2). The HRs by the patients’ place of residence are provided in Supplementary Table 2.

**Table 2:** Cox regression analysis to evaluate factors on the incidence of fractures.

Variable	HR for fractures [95% CI]	*p*-value
Age	1.062 [1.060–1.064]	< 0.001*
Sex (women = 1)	0.914 [0.824–1.015]	0.092
Prescriptions before entry		
BPs	1.153 [1.075–1.237]	< 0.001*
PTH	1.734 [1.605–1.872]	< 0.001*
Menatetrenone	1.167 [1.047–1.300]	0.005
SERMs	1.089 [1.014–1.170]	0.019
Incident fractures before entry		
Hip	1.054 [0.783–1.418]	0.731
Vertebra	1.888 [1.723–2.065]	< 0.001*
Radius	0.000 [0.000–over 10^10^]	0.960
Treatment	0.936 [0.904–0.970]	< 0.001*

HRs by the patients’ place of residence (prefectures) are provided in Supplementary Table 2.

*CI* confidence interval.

*p < 0.001 is considered significant.

**Figure 3:**
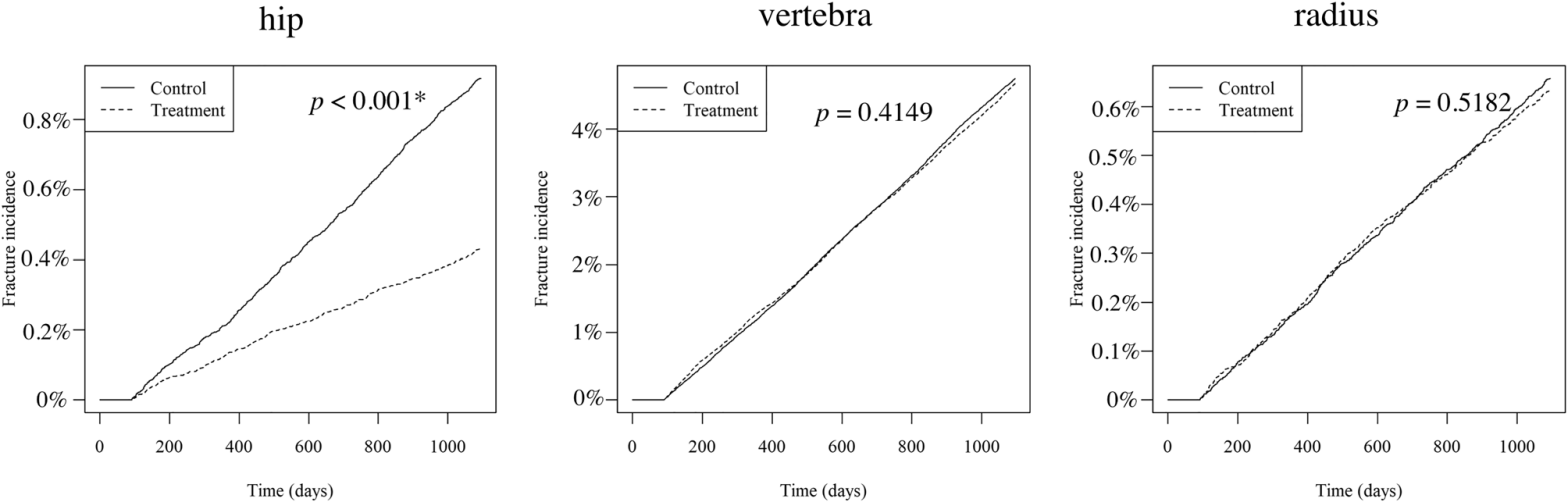
Cumulative incidence of fractures by site. *p < 0.001 is considered significant.

Fracture rates were compared by site. The site-specific fracture incidence rates were 0.89% (938 cases) and 0.42% (441 cases) (hip), 4.71% (4,952 cases) and 4.65% (4,883 cases) (vertebrae), and 0.64% (672 cases) and 0.62% (650 cases) (radius) (Fig. 3). The log-rank test showed a significant difference only for the hips (*p* < 0.001*) and no significant difference for the vertebrae and radius (*p* = 0.4149 and *p* = 0.5182, respectively).

**Figure 4:**
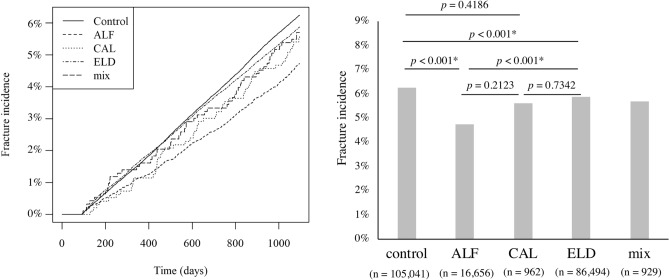
Cumulative incidence of total fractures by the type of vitamin D. *p < 0.001 is considered significant.

Subgroup analyses were performed for each type of administered vitamin D. The ALF, CAL, ELD, and mix groups consisted of 16,656, 962, 86, 494, and 929 patients, respectively. The fracture incidence of these groups was compared with that of the PS-matched control group in the main analysis (n = 105,041). The Kaplan–Meier curve and the total fracture rate of these five groups are shown in Fig. 4. Total fracture rates were 6.25% (6562 cases), 4.73% (788 cases), 5.61% (54 cases), 5.87% (5079 cases), and 5.71% (53 cases) in the control, ALF, CAL, ELD, and mix groups, respectively. The ALF and ELD groups demonstrated significantly lower fracture rates compared to the control group, and the ALF group demonstrated significantly lower fracture rate than the ELD group (*p* < 0.001*). No significant difference was observed between the other groups. Table 3 presents the characteristics of the subgroups. In the ELD group, the age was significantly younger, the proportion of patients prescribed BPs before entry was significantly higher, and the proportion of patients prescribed PTH, menatetrenone, and SERMs was significantly lower compared to the ALF group. Additionally, the proportion of patients who experienced hip and vertebral fractures before entry was significantly lower (*p* < 0.001*). After controlling for age, sex, prescriptions before entry, incident fractures before entry, and place of residence using a Cox regression analysis, the HRs for the incidence of new fractures in relation to the type of vitamin D are presented in Table 4. The HR showed a significant reduction only in the ALF group (HR 0.676 [95% CI 0.628–0.728]).

**Table 3:** Subgroups of the PS-matched treatment group by the type of vitamin D.

	ALF group	CAL group	ELD group	Mix group	*p*-value
n	16,656	962	86,494	929	
Age	75.5 ± 8.6	75.4 ± 8.9	73.0 ± 8.6	76.9 ± 9.0	< 0.001*
Sex					
Men	393 (2.4%)	27 (2.8%)	1922 (2.2%)	31 (3.3%)	0.058
Women	16,263 (97.6%)	935 (97.2%)	84,572 (97.8%)	898 (96.7%)	
Prescriptions before entry					
BPs	6043 (36.3%)	353 (36.7%)	42,165 (48.7%)	366 (39.4%)	< 0.001*
PTH	882 (5.3%)	26 (2.7%)	3469 (4.0%)	72 (7.8%)	< 0.001*
Menatetrenone	690 (4.1%)	68 (7.1%)	1566 (1.8%)	48 (5.2%)	< 0.001*
SERMs	8765 (52.6%)	509 (52.9%)	43,909 (50.8%)	473 (50.9%)	< 0.001*
Incident fractures before entry (hip + vertebra + radius)	484 (2.9%)	18 (1.9%)	1152 (1.3%)	55 (5.9%)	< 0.001*

Age is expressed as mean ± standard deviation.

*SMD* standardized mean difference.

*p < 0.001 is considered significant.

**Table 4:** The HRs for the incidence of fractures in relation to the type of vitamin D.

Variable	HR for fractures [95% CI]	*p*-value
The type of vitamin D		
ALF	0.676 [0.628–0.728]	< 0.001*
CAL	0.817 [0.625–1.068]	0.139
ELD	1.003 [0.967–1.040]	0.881
Mix	0.692 [0.528–0.907]	0.008

Age, sex, prescriptions before entry, incident fractures before entry, and place of residence (prefectures) were adjusted (data not shown).

*CI* confidence interval.

*p < 0.001 is considered significant.

## Discussion

This study demonstrated an association between vitamin D and a decrease in fracture incidence using PS matching. By site, the incidence of hip fractures significantly decreased, but vertebral and radius fractures did not show a significant reduction. The decrease in total fractures was primarily due to the decrease in hip fractures. These results are consistent with previous studies showing that vitamin D reduced the incidence of fractures. Since all national data are registered in the NDB, this study is considered free of selection bias.

In subgroup analyses, both the ELD and ALF groups had a lower incidence rate of total fractures than the PS-matched control group. However, the incidence rate of fractures was significantly higher in the ELD group compared to the ALF group (Fig. 4). Furthermore, the HRs for new fractures were reduced only in the ALF group after the Cox regression analysis (Table 4). This finding is inconsistent with a meta-analysis showing that ELD had a higher effect on bone density increase and fracture prevention than ALF^20^. This is possible because the present study is not an RCT but a retrospective study. Although PS matching partially adjusted for background differences between the control and treatment groups, patient characteristics differed between the ALF and ELD groups. For example, the average age of the ELD group was significantly lower than that of the AFL and CAL groups, although age was positively related to the incidence of fractures (Table 3). This suggests that the characteristics of patients administered ELD might be different from that of patients administered ALF or CAL. ELD was more recently available than ALF and CAL, is reported to be more effective in the treatment of osteoporosis^17–20^, thus might have been administered to patients at particularly high risk of fractures. In addition, inconsistent with previous studies that suggested that vitamin D analogs reduced vertebral fractures^14, 15^, vitamin D did not reduce the risk of vertebral fractures (Fig. 3), which might be considered a limitation of PS matching.

While active forms of vitamin D reduced the risk of developing fractures compared with vitamin D supplementation^14^, vitamin D supplementation could reduce the fracture risk, especially in combination with calcium^13^. In Japan, vitamin D supplements are available over-the-counter, but active forms of vitamin D are not. Patients in the control group are guaranteed not to receive active forms of vitamin D, but they might have been taking vitamin D supplements, which could have affected the results.

In the subanalysis, the prescription of CAL was not associated with a lower incidence of fractures. Only 962 patients in the PS-matched treatment group were prescribed CAL, suggesting that CAL is rarely prescribed in Japan. CAL did not reduced the incidence of vertebral fractures^15^, while it increased bone mineral density at the lumbar spine in osteoporotic patients with vitamin D deficiency^29^. The incidence of fractures in the CAL group was 5.61%, which was slightly lower than the 5.71% in the ELD group, suggesting the statistical power was insufficient.

Although treatment of osteoporosis is important to prevent the initial hip fracture, older people may have difficulty using other drugs (e.g., BPs) due to low activities of daily living, comorbidities, and social circumstances, and active vitamin D could be a treatment option.

This study has several limitations. Firstly, although we performed PS matching, it was not possible to adjust for all patients background. We adjusted for age, sex, prescriptions of osteoporosis medications before entry, incident fractures before entry, and regions of the patients, but could not adjust for other possible confounders that could affect the risk of fractures. For instance, BMD data is not included in the NDB, and information on other confounding factors (e.g., activities of daily living, exercise habits, dietary habits, body mass index, socioeconomic status, living situation, etc.) is also unavailable from the NDB, and their effects could not be taken into account. Secondly, we excluded patients who were prescribed non-osteoporotic drugs. This was intended to examine the association between vitamin D and the incidence of fractures after excluding the possible influence of other diseases as much as possible. However, since the remaining study population could be different from older people in general (often characterized by multimorbidity and polypharmacy), results of the present study might not be applicable to them. Furthermore, not being on medication does not necessarily mean not having the diseases. We could not consider the possible effects of comorbidities and medical histories on the risk of developing new fractures (e.g., diabetes mellitus, rheumatoid arthritis, etc.). However, due to the restrictions of the NDB, this was the limit of the patient information that could be obtained. Finally, data of the patients who died within 3 years of entry were not extracted from the beginning, which might have affected the result. Due to these major limitations and the fact that this is not an RCT, the causal relationship between vitamin D administration and reduced incidence of fractures cannot be assessed.

In summary, the results of this study suggest that vitamin D was associated with lower incidence of hip fractures. Reevaluation of the usefulness of vitamin D may lead to the development of osteoporosis treatment for older people who require individualized care.

### Supplementary Information

Supplementary Information.

Supplementary Table 1.

Supplementary Table 2.

## Data Availability

The data that support the findings of this study are available from the MHLW but restrictions apply to the availability of these data, which were used under license for the current study, and so are not publicly available. Data are however available from Sumito Ogawa upon reasonable request and with permission of the MHLW.
