# Supplementary material for: Prescription of vitamin D was associated with a lower incidence of hip fractures
Source: Sci Rep. 2023 Aug 9;13:12889. doi: 10.1038/s41598-023-40259-6 (PMC10412563; doi:10.1038/s41598-023-40259-6)
Supplement: Supplementary file 2 — Supplementary Table 1. [file 41598_2023_40259_MOESM2_ESM.docx]

**Supplementary Table 1. The patients’ place of residence before and after the PS matching.**

|  | Before PS matching | | | | After PS matching | | |
| --- | --- | --- | --- | --- | --- | --- | --- |
|  | Untreated patients | Treated patients | SMD | *p*-value | PS-matched control group | PS-matched treatment group | SMD |
| n | 422,454 | 169,774 |  |  | 105,041 | 105,041 |  |
| Prefectures (codes) |  | | | |  | | |
| Hokkaido (1)  Aomori (2)  Iwate (3)  Miyagi (4)  Akita (5)  Yamagata (6)  Fukushima (7)  Ibaraki (8)  Tochigi (9)  Gunma (10)  Saitama (11)  Chiba (12)  Tokyo (13)  Kanagawa (14)  Niigata (15)  Toyama (16)  Ishikawa (17)  Fukui (18)  Yamanashi (19)  Nagano (20)  Gifu (21)  Shizuoka (22)  Aichi (23)  Mie (24)  Shiga (25)  Kyoto (26)  Osaka (27)  Hyogo (28)  Nara (29)  Wakayama (30)  Tottori (31)  Shimane (32)  Okayama (33)  Hiroshima (34)  Yamaguchi (35)  Tokushima (36)  Kagawa (37)  Ehime (38)  Kochi (39)  Fukuoka (40)  Saga (41)  Nagasaki (42)  Kumamoto (43)  Oita (44)  Miyazaki (45)  Kagoshima (46)  Okinawa (47) | 17,371 (4.1%)  5,539 (1.3%)  5,585 (1.3%)  7,560 (1.8%)  5,946 (1.4%)  4,674 (1.1%)  6,605 (1.6%)  8,745 (2.1%)  5,934 (1.4%)  6,781 (1.6%)  18,574 (4.4%)  14,987 (3.5%)  32,683 (7.7%)  21,719 (5.1%)  8,885 (2.1%)  4,992 (1.2%)  5,626 (1.3%)  2,116 (0.5%)  2,953 (0.7%)  7,849 (1.9%)  6,664 (1.6%)  13,760 (3.3%)  21,043 (5.0%)  6,277 (1.5%)  4,431 (1.0%)  8,097 (1.9%)  22,335 (5.3%)  14,785 (3.5%)  3,435 (0.8%)  3,253 (0.8%)  2,384 (0.6%)  2,733 (0.6%)  7,131 (1.7%)  8,337 (2.0%)  4,831 (1.1%)  2,847 (0.7%)  3,182 (0.8%)  4,632 (1.1%)  1,980 (0.5%)  13,742 (3.3%)  2,371 (0.6%)  5,006 (1.2%)  4,896 (1.2%)  3,365 (0.8%)  3,838 (0.9%)  4,469 (1.1%)  2,632 (0.6%) | 7,218 (4.3%)  2,215 (1.3%)  1,690 (1.0%)  3,657 (2.2%)  1,665 (1.0%)  2,022 (1.2%)  3,024 (1.8%)  2,881 (1.7%)  2,222 (1.3%)  2,531 (1.5%)  6,881 (4.1%)  6,213 (3.7%)  12,044 (7.1%)  8,853 (5.2%)  4,108 (2.4%)  1,812 (1.1%)  2,099 (1.2%)  996 (0.6%)  1,087 (0.6%)  3,291 (1.9%)  2,356 (1.4%)  5,403 (3.2%)  8,427 (5.0%)  1,919 (1.1%)  1,332 (0.8%)  2,732 (1.6%)  11,766 (6.9%)  6,490 (3.8%)  1,816 (1.1%)  1,546 (0.9%)  661 (0.4%)  965 (0.6%)  2,259 (1.3%)  3,709 (2.2%)  1,652 (1.0%)  1,052 (0.6%)  1,182 (0.7%)  1,881 (1.1%)  1,150 (0.7%)  7,473 (4.4%)  805 (0.5%)  1,998 (1.2%)  2,435 (1.4%)  1,246 (0.7%)  1,559 (0.9%)  2,059 (1.2%)  1,497 (0.9%) | 0.007  0.001  0.031  0.026  0.039  0.008  0.017  0.027  0.008  0.009  0.017  0.006  0.025  0.003  0.021  0.011  0.008  0.012  0.007  0.006  0.016  0.004  0.001  0.031  0.028  0.023  0.069  0.017  0.027  0.015  0.025  0.010  0.029  0.015  0.017  0.007  0.007  0.001  0.028  0.060  0.012  0.001  0.024  0.007  0.001  0.015  0.030 | 0.015  0.853  <0.001*  <0.001*  <0.001*  0.006  <0.001*  <0.001*  0.004  0.001  <0.001*  0.037  <0.001*  0.251  <0.001*  <0.001*  0.004  <0.001*  0.014  0.040  <0.001*  0.144  0.785  <0.001*  <0.001*  <0.001*  <0.001*  <0.001*  <0.001*  <0.001*  <0.001*  0.001  <0.001*  <0.001*  <0.001*  0.020  0.021  0.712  <0.001*  <0.001*  <0.001*  0.804  <0.001*  0.014  0.732  <0.001*  <0.001* | 4,745 (4.5%)  1,373 (1.3%)  1,161 (1.1%)  2,456 (2.3%)  1,180 (1.1%)  1,019 (1.0%)  1,751 (1.7%)  1,998 (1.9%)  1,331 (1.3%)  1,346 (1.3%)  4,132 (3.9%)  3,924 (3.7%)  7,610 (7.2%)  5,397 (5.1%)  2,695 (2.6%)  1,011 (1.0%)  1,260 (1.2%)  693 (0.7%)  622 (0.6%)  2,143 (2.0%)  1,471 (1.4%)  3,753 (3.6%)  5,473 (5.2%)  1,089 (1.0%)  866 (0.8%)  1,586 (1.5%)  6,198 (5.9%)  3,787 (3.6%)  1,057 (1.0%)  794 (0.8%)  388 (0.4%)  609 (0.6%)  1,212 (1.2%)  2,129 (2.0%)  964 (0.9%)  535 (0.5%)  717 (0.7%)  1,141 (1.1%)  759 (0.7%)  4,738 (4.5%)  495 (0.5%)  1,121 (1.1%)  1,381 (1.3%)  779 (0.7%)  1,046 (1.0%)  1,244 (1.2%)  796 (0.8%) | 4,757 (4.5%)  1,418 (1.3%)  1,164 (1.1%)  2,471 (2.4%)  1,248 (1.2%)  1,083 (1.0%)  1,787 (1.7%)  2,079 (2.0%)  1,334 (1.3%)  1,344 (1.3%)  4,121 (3.9%)  4,045 (3.9%)  7,349 (7.0%)  5,921 (5.6%)  2,657 (2.5%)  1,082 (1.0%)  1,252 (1.2%)  644 (0.6%)  610 (0.6%)  2,083 (2.0%)  1,441 (1.4%)  3,835 (3.7%)  5,314 (5.1%)  1,119 (1.1%)  860 (0.8%)  1,554 (1.5%)  6,335 (6.0%)  3,732 (3.6%)  1,019 (1.0%)  811 (0.8%)  407 (0.4%)  664 (0.6%)  1,247 (1.2%)  2,170 (2.1%)  1,020 (1.0%)  631 (0.6%)  777 (0.7%)  1,105 (1.1%)  679 (0.6%)  4,898 (4.7%)  487 (0.5%)  1,130 (1.1%)  1,379 (1.3%)  823 (0.8%)  1,033 (1.0%)  1,249 (1.2%)  808 (0.8%) | 0.001  0.004  <0.001  0.001  0.006  0.006  0.003  0.006  <0.001  <0.001  0.001  0.006  0.010  0.022  0.002  0.007  0.001  0.006  0.001  0.004  0.002  0.004  0.007  0.003  0.001  0.003  0.006  0.003  0.004  0.002  0.003  0.007  0.003  0.003  0.006  0.012  0.007  0.003  0.009  0.007  0.001  0.001  <0.001  0.005  0.001  <0.001  0.001 |

**p* < 0.001 is considered significant.

SMD, standardized mean difference
