# Supplementary material for: Prescription of vitamin D was associated with a lower incidence of hip fractures
Source: Sci Rep. 2023 Aug 9;13:12889. doi: 10.1038/s41598-023-40259-6 (PMC10412563; doi:10.1038/s41598-023-40259-6)
Supplement: Supplementary file 3 — Supplementary Table 2. [file 41598_2023_40259_MOESM3_ESM.docx]

**Supplementary Table 2. The HRs for fractures by the patients’ place of residence (prefectures).**

| Variable | HR for fractures [95% CI] | *p*-value |
| --- | --- | --- |
| Prefectures  Hokkaido (1)  Aomori (2)  Iwate (3)  Miyagi (4)  Akita (5)  Yamagata (6)  Fukushima (7)  Ibaraki (8)  Tochigi (9)  Gunma (10)  Saitama (11)  Chiba (12)  Tokyo (13)  Kanagawa (14)  Niigata (15)  Toyama (16)  Ishikawa (17)  Fukui (18)  Yamanashi (19)  Nagano (20)  Gifu (21)  Shizuoka (22)  Aichi (23)  Mie (24)  Shiga (25)  Kyoto (26)  Osaka (27)  Hyogo (28)  Nara (29)  Wakayama (30)  Tottori (31)  Shimane (32)  Okayama (33)  Hiroshima (34)  Yamaguchi (35)  Tokushima (36)  Kagawa (37)  Ehime (38)  Kochi (39)  Fukuoka (40)  Saga (41)  Nagasaki (42)  Kumamoto (43)  Oita (44)  Miyazaki (45)  Kagoshima (46)  Okinawa (47) | 0.873 [0.791-0.963]  0.772 [0.650-0.916]  0.751 [0.627-0.899]  0.783 [0.686-0.893]  0.700 [0.583-0.841]  0.755 [0.625-0.912]  0.582 [0.492-0.688]  0.831 [0.723-0.955]  0.831 [0.703-0.982]  0.787 [0.665-0.931]  0.747 [0.668-0.835]  0.794 [0.712-0.886]  0.769 [0.704-0.841]  0.892 [0.813-0.979]  0.782 [0.690-0.885]  0.717 [0.590-0.871]  0.891 [0.755-1.051]  0.872 [0.701-1.083]  0.734 [0.574-0.939]  0.791 [0.691-0.907]  0.776 [0.659-0.913]  0.885 [0.796-0.983]  0.778 [0.705-0.859]  0.812 [0.677-0.974]  0.671 [0.535-0.841]  0.806 [0.689-0.942]  0.886 [0.810-0.970]  0.902 [0.811-1.002]  0.784 [0.647-0.950]  0.728 [0.586-0.904]  0.945 [0.725-1.232]  1.090 [0.894-1.329]  0.931 [0.792-1.095]  0.900 [0.792-1.023]  0.887 [0.741-1.061]  1.080 [0.870-1.340]  0.865 [0.707-1.058]  0.964 [0.820-1.133]  0.872 [0.707-1.076]  0.991 [0.902-1.090]  1.170 [0.939-1.457]  0.865 [0.730-1.025]  1.026 [0.889-1.184]  1.031 [0.859-1.237]  1.028 [0.873-1.210]  1.117 [0.967-1.292]  0.952 [0.781-1.162] | 0.007  0.003  0.002  <0.001*  <0.001*  0.004  <0.001*  0.009  0.030  0.005  <0.001*  <0.001*  <0.001*  0.017  <0.001*  <0.001*  0.170  0.215  0.014  <0.001*  0.002  0.023  <0.001*  0.025  <0.001*  0.007  0.009  0.055  0.013  0.004  0.676  0.393  0.388  0.108  0.190  0.486 0.158  0.657  0.201  0.859  0.161  0.095  0.725  0.743  0.742  0.134  0.631 |

CI, confidence interval. **p* < 0.001 is considered significant.
